# Enhancing intelligence source performance management through two-stage stochastic programming and machine learning techniques

**DOI:** 10.3389/fdata.2025.1640539

**Published:** 2025-09-22

**Authors:** Lucas Wafula Wekesa, Stephen Korir

**Affiliations:** Strathmore Institute of Mathematical Sciences, Strathmore University, Nairobi, Kenya

**Keywords:** intelligence performance, stochastic programming, machine learning, intelligence source evaluation, operational uncertainty, HUMINT performance management, behavioral risk prediction

## Abstract

**Introduction:**

The effectiveness of intelligence operations depends heavily on the reliability and performance of human intelligence (HUMINT) sources. Yet, source behavior is often unpredictable, deceptive or shaped by operational context, complicating resource allocation and tasking decisions.

**Methods:**

This study developed a hybrid framework combining Machine Learning (ML) techniques and Two-Stage Stochastic Programming (TSSP) for HUMINT source performance management under uncertainty. A synthetic dataset reflecting HUMINT operational patterns was generated and used to train classification and regression models. The extreme Gradient Boosting (XGBoost) and Support Vector Machines (SVM) were applied for behavioral classification and prediction of reliability and deception scores. The predictive outputs were then transformed into scenario probabilities and integrated into the TSSP model to optimize task allocation under varying behavioral uncertainties.

**Results:**

The classifiers achieved 98% overall accuracy, with XGBoost exhibiting higher precision and SVM demonstrating superior recall for rare but operationally significant categories. The regression models achieved R-squared scores of 93% for reliability and 81% for deception. These predictive outputs were transformed into scenario probabilities for integration into the TSSP model, optimizing task allocation under varying behavioral risks. When compared to a deterministic optimization baseline, the hybrid framework delivered a 16.8% reduction in expected tasking costs and a 19.3% improvement in mission success rates.

**Discussion and conclusion:**

The findings demonstrated that scenario-based probabilistic planning offers significant advantages over static heuristics in managing uncertainty in HUMINT operations. While the simulation results are promising, validation through field data is required before operational deployment.

## 1 Introduction

### 1.1 Background of the study

The performance of intelligence sources remains a cornerstone in the effectiveness of modern intelligence operations. Whether drawn from human contacts, signals, open-source materials, or technical surveillance, the value of intelligence is deeply tied to the reliability, timeliness, and accuracy of its source (Lowenthal, 2020). Intelligence practitioners are routinely required to assess the strength and credibility of their sources under rapidly shifting operational conditions (Robson and Chen, 2022). However, current approaches to evaluating these sources often depend on static scoring models or qualitative judgments, many of which cannot capture uncertainty or respond to dynamic shifts in source behavior (Clark, 2013). Operationally, these limitations can lead to significant inefficiencies. Intelligence officers and organizations may continue investing resources in sources that no longer yield actionable or trustworthy information (Bose, 2008). At the same time, emerging or underutilized sources that exhibit high performance may be overlooked. This misallocation is compounded by the fact that intelligence gathering frequently occurs in environments marked by deception, misinformation, and adversarial manipulation (Zegart, 2022). In such contexts, decision-makers must rely on structured, data-informed strategies to mitigate operational risks and maximize the value of their assets.

In addressing this problem, advanced computational methods are gaining traction in intelligence performance management. A two-stage stochastic programming (TSSP) allows planners to model uncertainty by structuring decisions in two phases: first, by allocating resources before full information is available, and second, by adjusting those decisions once uncertainty resolves (Birge and Louveaux, 2011). This is especially relevant in intelligence, where early decisions often must be made with incomplete knowledge about source effectiveness. Simultaneously, machine learning (ML) provides a mechanism to detect patterns in historical reporting, allowing analysts to classify sources as high or low performing based on prior performance indicators such as corroboration, timeliness, or contextual value (Jordan and Mitchell, 2015; Phythian, 2020). ML models learn from past trends, and they can support predictive assessments that complement the forward-looking uncertainty management of TSSP models.

This study offers a different approach. It proposes a hybrid framework that combines the predictive capabilities of machine learning with the uncertainty modeling power of two-stage stochastic programming. The goal is threefold, intending to provide an adaptive, data-informed system that, first, assesses and predicts human intelligence source performance; second, optimizes the allocation of scarce intelligence resources under uncertainty; and third, minimizes the operational risks associated with source misjudgment. Therefore, the objective of this study is to develop an integrated analytical framework that enhances the performance management of human intelligence sources by combining machine learning techniques with two-stage stochastic programming under conditions of uncertainty.

## 2 Literature review

### 2.1 Historical and theoretical foundations of HUMINT source evaluation and tasking

HUMINT has long been a central pillar of intelligence operations, particularly in contexts requiring cultural insights, strategic access, or ground-level information (Yankov, 2019). Traditionally, the evaluation of HUMINT sources has relied on subjective judgments, emphasizing analysts' experience and behavioral impressions, which can lead to inconsistencies and biases in assessments (Johnson, 2010). According to (Clark 2013), this approach is rooted in Cold War-era tradecraft, where qualitative factors such as motivation, access, and personality traits were considered during agent recruitment and tasking. Further, the theoretical foundation for HUMINT evaluation borrows from fields such as psychology, decision theory, and counterintelligence. For instance, Heuer's work on the psychology of intelligence analysis emphasizes the cognitive processes involved in making judgments under conditions of uncertainty and ambiguity, drawing from cognitive psychology to enhance the understanding of how analysts interpret incomplete information and the dangers of over-reliance on intuitive reasoning (Heuer, 1999).

However, more recent work stresses the need for structured analytic techniques. For instance, (Clark 2013) proposed a target-centric model that advocates for continuous feedback between analysts and sources, emphasizing interdependence and responses in intelligence gathering. However, many of these models assume ideal tasking environments. In reality, source reliability is fluid, influenced by changing personal motivations, external pressure, and operational risks (Willard et al., 2022). As such, static assessments often fail to reflect the evolving performance of a source. Equally, (Treverton and Gabbard 2008) indicates that despite advances in structured tradecraft, there remains a significant gap in scalable, adaptive frameworks that account for uncertainty, deception, and real-time variability in HUMINT behavior.

### 2.2 Review of TSSP applications in operations research and military decision-making under uncertainty

TSSP has become a well-established method in operations research for addressing decision-making under uncertainty, especially in sectors where risks, costs, and timing are interdependent (Badejo and Ierapetritou, 2025). Within the military and defense environments, the relevance of TSSP is pronounced due to the inherently unpredictable nature of adversarial behavior, supply chain disruption, and battlefield conditions (Nissen et al., 2018).

The conceptual foundation of TSSP lies in its two-stage decision-making structure. As illustrated by (Birge and Louveaux 2011), the first stage involves decisions made before uncertainty is revealed, while the second stage captures corrective actions after uncertain events are observed. This design enables decision-makers to preemptively plan for contingencies without requiring perfect information. Mathematically, TSSP allows the optimisation of an expected value objective function under probabilistic constraints, making it a suitable choice in planning domains that demand flexibility. The value of such a framework in high-risk environments is well-supported in the literature. (Shapiro et al. 2021) emphasizes that TSSP models outperform deterministic models in environments characterized by incomplete or probabilistic data, particularly when the cost of failure is high. For instance, in defense logistics, the difference in cost between robust TSSP-based and deterministic planning has been found to range between 15% and 30% in efficiency gains, especially in time-sensitive operations (Bodur and Luedtke, 2017).

The first application of the TSSP model was in military logistics and deployment by the U.S. Department of Defense, which used stochastic optimisation for pre-positioning supplies in anticipation of deployment across multiple geopolitical zones (Li et al., 2022). In these models, first-stage decisions included the allocation of fuel, ammunition, and food to bases, while second-stage adjustments respond to emergent scenarios such as geopolitical tensions or natural disasters (Bodur and Luedtke, 2017). Similarly, in weapon system deployment, (Schneider et al., 2014) applied TSSP to optimize spare part provisioning for unmanned aerial vehicles (UAVs), where mission-critical failures and maintenance delays are uncertain. Their model reduced logistical downtime by 19%, demonstrating the value of scenario-based planning in enhancing equipment availability in a volatile operating environment. In recent years, researchers have used TSSP to manage the repositioning of military supplies across uncertain demand locations. For instance, (Bodaghi and Othman 2020) demonstrated that TSSP could improve logistics readiness for peacekeeping operations by accounting for variability in regional stability, transport accessibility, and sudden surge demands. Their results showed a reduction of unmet demand by over 25%, compared to deterministic methods.

Despite these successes, the application of TSSP to intelligence resource management, especially human intelligence (HUMINT), remains sparse. Intelligence operations often face the same fundamental structure of uncertainty; analysts and officers must allocate attention, time, or funding to sources without knowing their true future reliability (Neequaye and Granhag, 2023). However, existing resource decisions in intelligence are more likely to rely on heuristic or subjective assessments than structured optimisation models. This gap is critical. As intelligence organizations accumulate more structured data on source behavior, such as timeliness, corroboration, and response rates, opportunities emerge for model-based planning that is both probabilistic and adaptive.

### 2.3 Machine learning in intelligence and behavioral modeling: deception detection, trust scoring, anomaly detection

The application of ML in intelligence analysis has grown rapidly, particularly in the modeling of complex human behaviors that are difficult to detect through manual analysis. Recent studies emphasize its growing utility in three core areas relevant to intelligence source evaluation: deception detection, trust scoring, and anomaly detection. These domains leverage large volumes of operational or behavioral data to support more objective, adaptive, and scalable intelligence assessments.

In the domain of deception detection, (Fornaciari et al. 2020) developed a model using generative language techniques to detect deceptive content in user-generated reviews. Although their study was situated in the context of online consumer data, their approach applies to intelligence analysis, where linguistic deception is a common tactic. Their model utilized patterns in syntax, sentiment, and language complexity to identify falsehoods, achieving significant predictive accuracy. Similarly, (Ott et al. 2013) demonstrated the effectiveness of Support Vector Machines and linguistic feature engineering in identifying deceptive opinion spam, reporting classification accuracies above 85% on benchmark datasets. These studies highlight the value of supervised learning models in flagging potentially false or manipulated information based on subtle language cues; tools that could be adapted for intelligence source validation.

Trust scoring has also been the subject of recent scholarly focus. (Kaplan et al. 2021) conducted a meta-analysis of trust modeling in artificial intelligence and found that data-driven trust scores, calculated using prior success, corroboration history, and behavioral consistency, are increasingly viable in human-machine and human-human interaction assessments. Their findings support the integration of trust scoring into intelligence workflows, where algorithms may assess sources based on objective performance indicators. Complementing this, (Lee and See 2004) emphasized the importance of designing algorithms that foster appropriate trust in automated systems, noting that excessive trust can be as dangerous as mistrust in high-stakes environments. Their findings suggest that ML-based scoring systems must remain transparent and interpretable, particularly in life-threatening intelligence operations.

In anomaly detection, (Ahmed et al. 2016) conducted a comprehensive review of unsupervised ML techniques to identify behavioral or network anomalies. Their studies cataloged algorithms such as k-means clustering, DBSCAN, and Isolation Forests, all of which have been used to identify deviations from expected patterns in large datasets. In the context of intelligence operations, such methods are useful for detecting shifts in the reporting style of a source, timing irregularities, or abnormal access behaviors that might indicate compromise or coercion. (Pérez-Rosas et al. 2018) further contributed to this domain by applying deep learning to the detection of fake news, demonstrating how models trained on contextual and linguistic inputs can signal abnormal or disingenuous content with high precision.

### 2.4 Existing efforts and limitations in automating intelligence source management

In recent years, intelligence organizations have adopted various digital tools to enhance the tracking and evaluation of source performance. These include structured scorecards, reporting dashboards, and centralized digital repositories that collect metadata such as tasking frequency, report timeliness, and corroboration status (Lowenthal, 2020). The tools typically track metadata such as report frequency, tasking responsiveness, source category, and reporting format, providing a structured approach to organizing and auditing intelligence activities.

However, (Zegart 2022) observes that despite advances in artificial intelligence and machine learning developments, the degree of true automation in source management remains limited. A major reason for this is the primary constraints in the sensitive and sparse nature of intelligence data. Unlike commercial datasets, which tend to be clean, labeled, and abundant, intelligence source information is often classified, fragmented, and compartmentalized, making it difficult to build statistically generalizable models (Wired Staff, 2022). Moreover, access to labeled data, necessary for machine learning algorithms, is often restricted or inconsistent across operational units. Consequently, according to (D'Amour et al. 2020) and (Tlamelo et al. 2021), prior ML-only approaches rely on stable historical patterns and perform well on clean, benchmark datasets, but they falter in operational settings marked by uncertainty, missing data, or domain shifts because of under-specified models that fail to generalize beyond the training environment.

(Rudin 2019) postulates that the institutional reluctance to delegate high-stakes decisions to algorithmic systems is another obstacle, particularly when the outputs affect source validation, operational trust, or legal responsibility. He argues for the avoidance of opaque black-box models in favor of interpretable machine learning systems, especially in critical domains like law enforcement, national security, and healthcare. This is echoed by (Danks and London 2017), who contend that explainability, fairness, and accountability must be embedded into the design of AI systems used in sensitive policy domains. In intelligence environments, where algorithmic decisions may have strategic and ethical implications, the preference for human-in-loop models remains strong (Park, 2023). As a result, even well-performing ML models are frequently relegated to advisory roles rather than being integrated into core performance management workflows.

### 2.5 Identified gaps and contribution of this study to the literature

The reviewed literature demonstrates that while conceptual models of source assessment and scoring exist, most remain static, heuristic-driven, and dependent on subjective judgments. As noted by (Lowenthal 2020), traditional methods rely on retrospective analysis, with minimal capacity for forward-looking planning or scenario modeling. Even with digitisation efforts, intelligence agencies have yet to adopt frameworks that incorporate behavioral uncertainty and probabilistic reasoning into source tasking decisions. In parallel, the operations research community has made significant advances in TSSP for logistics, deployment, and homeland security applications (Chen et al., 2023, 2024). These models have proven effective in contexts where decisions must be made under incomplete information, with contingency actions embedded into planning. However, the literature reveals a clear lack of application of TSSP models in intelligence-specific workflows, particularly in managing HUMINT sources, where behavior, reliability, and risk are inherently probabilistic. To date, there are no known studies that have combined TSSP with intelligence source evaluation to support adaptive tasking under uncertainty.

Furthermore, while machine learning has been successfully applied to behavioral modeling in intelligence, particularly for deception detection, anomaly identification, and trust-scoring (Fornaciari et al., 2020; Pérez-Rosas et al., 2018; Kaplan et al., 2021), its integration into operational decision-support systems remains limited. Current applications tend to operate in analytical silos, providing insights that are not directly actionable within resource allocation or planning systems (Van Puyvelde et al., 2017). The lack of integration between predictive models and optimisation frameworks results in missed opportunities to use real-time performance forecasts to guide resource distribution and strategic tasking decisions.

## 3 Methods and materials

### 3.1 Study design

This study adopts a mixed-methods research design, grounded in design science and decision-analytic modeling, to address the complex problem of source performance management in intelligence operations. The research integrates supervised machine learning for behavioral prediction and classification with TSSP for resource allocation under uncertainty. This mixed-method research design is suited to intelligence environments, where decision-makers face high-risk scenarios, limited verifiable information, and the need for adaptive planning based on evolving field realities (Lowenthal, 2020; Zegart, 2022). This design is justified because of the nature of the research problem, which requires both predictive modeling (to infer future source behavior) and prescriptive optimisation (to allocate resources effectively under uncertainty).

#### 3.1.1 Modeling intelligence tasking as a two-stage decision problem

At the core of the study is the formulation of the intelligence tasking problem as a TSSP. Let *x* ∈ ℝ^*n*^, represent vector of first-stage decision variables representing initial resource allocations to sources (such as funding, handler time, access to sensitive operations); ω ∈ Ω is the scenario representing a realization of uncertain source behavior (e.g., cooperative, deceptive, compromised), with associated probability *p*(ω); *y*(ω) ∈ ℝ^*m*^ is the second-stage (recourse) decision variables representing adaptive actions (e.g., re-tasking, disengagement, reinforcement); *c* ∈ ℝ^*n*^ be the cost vector for first-stage allocations; *q*(ω) ∈ ℝ^*m*^ be the cost vector for recourse actions under scenario ω; *A* ∈ ℝ^*r* × *n*^: constraint matrix for first-stage decisions; *T*(ω) ∈ ℝ^*s* × *n*^, *W*(ω) ∈ ℝ^*s* × *m*^ represent the constraint matrices for second-stage feasibility conditions and; *h*(ω) ∈ ℝ^*s*^ represent right-hand side of the second-stage constraints under scenario ω. The two-stage stochastic programming (TSSP) model is given by:


(1) $$\begin{array}{l} \underset{x}{\min}\left\{c^{⊤}x+{𝔼}_{\omega }\left[Q\left(x,\omega \right)\right]\right\} \end{array}$$

(2) $$\begin{array}{l} \text{subject to}\;Ax\leq b \end{array}$$

where the second-stage value function *Q*(*x*, ω) is:


(3) $$\begin{array}{l} Q\left(x,\omega \right)=\underset{y\left(\omega \right)}{\min}\left\{q{\left(\omega \right)}^{⊤}y\left(\omega \right)\right\} \end{array}$$

(4) $$\begin{array}{l} \text{subject to}\;T\left(\omega \right)x+W\left(\omega \right)y\left(\omega \right)\geq h\left(\omega \right) \end{array}$$

(5) $$\begin{array}{l} y\left(\omega \right)\geq 0 \end{array}$$

This formulation supports flexible planning, enabling the system to adapt to new behavioral outcomes, for instance, sources turning unreliable or exhibiting anomalous behavior, while controlling for expected operational costs and resource efficiency (Birge and Louveaux, 2011).

#### 3.1.2 Incorporating behavioral uncertainty through machine learning

To generate the scenario tree Ω, this study employs supervised ML models trained on historical and simulated intelligence data. The behavior of each source is predicted based on engineered features such as task completion rate, reporting frequency and delays, deception from linguistic and behavioral cues, handler feedback, and counterintelligence flags. Let $X_{i}\in {\mathbb{R}}^{d}$ represent the feature vector for source *i*, and *y*_*i*_ ∈ {0, 1} represent the binary label indicating whether the source is reliable (1) or not (0). The predictive machine learning model is defined as:


(6) $$\begin{array}{l} {ŷ}_{i}=f_{\theta }\left(X_{i}\right) \end{array}$$

where *f*_θ_ is the model parameterized by θ, trained to minimize the empirical loss:


(7) $$\begin{array}{l} \underset{\theta }{\min}\frac{1}{N}\overset{N}{\underset{i=1}{\sum }}L\left(f_{\theta }\left(X_{i}\right),y_{i}\right) \end{array}$$

The predicted probability of success, denoted π_*i*_, is then used to assign scenario probabilities in the TSSP model. For each scenario ω_*i*_, the scenario probability is defined as:


(8) $$\begin{array}{l} p\left(\omega_{i}\right)=\pi_{i}\cdot \left(1-r_{i}\right) \end{array}$$

where *r*_*i*_ ∈ [0, 1] is a deception risk score obtained through Natural Language Processing (NLP) or anomaly detection models. This integration transforms the machine learning predictions into actionable probabilistic inputs for the stochastic optimisation model (Rudin, 2019; Kaplan et al., 2021).

#### 3.1.3 Sequential decision-making logic

**Figure 1:**
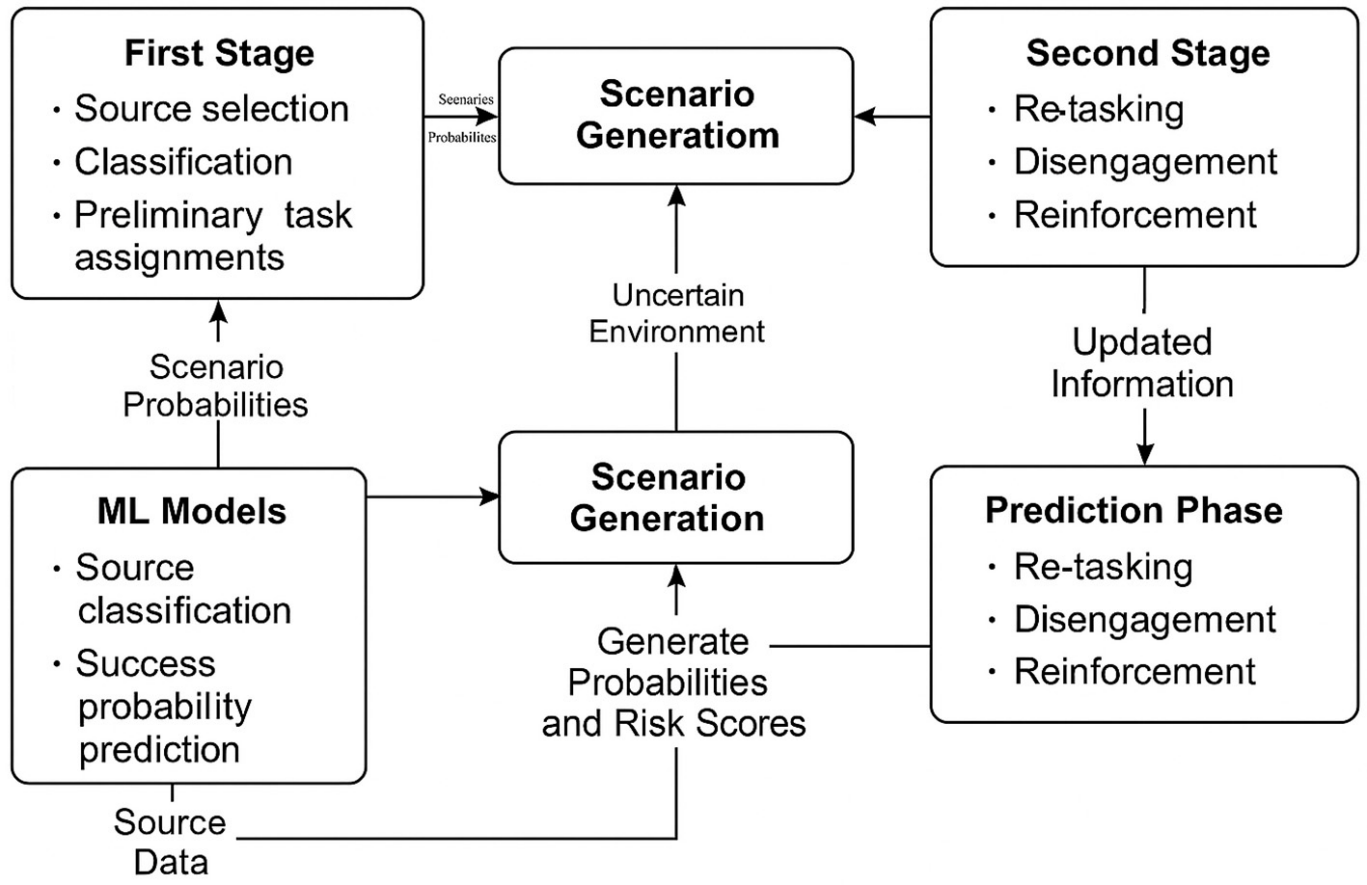
Proposed mixed-method framework integrating ML predictions with TSSP for intelligence source performance management.

Figure 1 shows the diagrammatic representation of the mixed-method framework.

This dual structure enables the intelligence decision-making process to be modeled sequentially, capturing both foresight and adaptability. In the first stage, strategic tasking decisions are made based on ML predictions of source reliability. In the second stage, as outcomes unfold or real-time updates are received, tactical adjustments such as reallocation, withdrawal, or reinforcement are made. This aligns with realistic command-and-control dynamics in national security operations where early decisions must be made under uncertainty but revised as ground intelligence evolves (Bodur and Luedtke, 2017).

### 3.2 Population and sampling techniques

Given the classified and ethically sensitive nature of operational intelligence data, this study does not utilize primary human subject data. Instead, the research focuses on a simulation-based sampling approach, informed by intelligence field structures and grounded in realistic operational dynamics. Since access to live or classified HUMINT performance data is restricted, this research generates a controlled, feature-rich synthetic dataset that emulates real-world source behavior under various operational conditions. This dataset serves both machine learning and stochastic optimisation scenario modeling.

#### 3.2.1 Target population and operational framing

The conceptual population consists of operational intelligence sources managed by a security agency. Each source is treated as a decision-making entity whose behavior impacts the success of intelligence-gathering missions. The population is designed to reflect sources with diverse risk profiles, engagement histories, and operational contexts. Attributes such as task responsiveness, deception tendencies, handler evaluations, and corroboration levels are captured to reflect real-world constraints and decision variables (see Table 1). The operational framing of this synthetic population is guided by relevant scholarly materials like the work advanced by (Lowenthal 2020) and (Zegart 2022), as well as existing simulation practices in security and defense modeling discussed by (Treverton and Gabbard 2008).

**Table 1:** Description of variables used in source performance analysis.

**Variable**	**Description**	**Type**
Tasking Frequency	Number of tasks assigned over a period	Discrete
Task success rate	Percentage of tasks completed successfully	Continuous
Report timeliness	Average lag between assignment and report submission	Continuous
Corroboration index	Extent to which reports align with other sources	Ratio
Deception score	NLP/ML-derived indicator of misleading or suspicious reporting	Probabilistic
Handler confidence rating	Qualitative assessment of source performance (ordinal scale)	Ordinal
Counterintelligence alert	Binary flag for suspected compromise or hostile influence	Categorical
Source behavior category	Classification for training (e.g., cooperative, deceptive, dormant)	Categorical

These features are mapped to model both first-stage decision variables, for instance, tasking allocation, and second-stage recourse decisions such as disengagement and reinforcement within the TSSP model.

#### 3.2.2 Behavior classification for simulation

The study assigns a behavioral class to each of the simulated sources as shown in Table 2 to enrich the behavioral diversity and improve model generalizability. These categories help segment the population for training supervised learning models and structuring optimisation scenarios. These categories align with known HUMINT operational profiles and are referenced in literature on behavioral modeling in intelligence operations (Fornaciari et al., 2020; Kaplan et al., 2021).

**Table 2:** Behavioral categories of intelligence sources.

**Category**	**Operational description**	**Strategic implication**
Cooperative	Compliant, consistent, truthful in reporting	Prioritized for core intelligence operations
Deceptive	Deliberately misleading or manipulative	High risk; monitored for misinformation threats
Coerced	Performs under external pressure or blackmail	Unstable; assessed for task suitability
Dormant	Non-responsive, inactive under tasking	Considered for disengagement
Compromised	Suspected or confirmed to be working with hostile actors	Immediate operational and counterintelligence response

#### 3.2.3 Sampling strategy and simulation size

A purposive sampling strategy was used to ensure behavioral representativeness and operational realism. The study simulates two synthetic datasets, each serving a function within the hybrid framework. The first dataset is a large sample of 5,000 records used for training and validating the machine learning models. This size balances computational efficiency with the need for behavioral and contextual diversity. Prior work in classification and behavioral modeling suggests that datasets ranging from 3,000 to 10,000 instances are suitable for capturing sufficient variation without overfitting or under-sampling rare cases (Gomez-Uribe and Hunt, 2015; Ribeiro et al., 2016). Further, behavioral profiles were parameterised based on established literature on HUMINT performance variability, including traits such as reliability shifts, deception risk, corroboration frequency, and reporting timeliness (Treverton and Gabbard, 2008; Matovski, 2020). Each synthetic source was modeled across a multi-dimensional feature space incorporating quantitative indicators (e.g., task success rates) and categorical attributes (e.g., operation role, exposure level). This structure aimed to simulate realistic performance patterns under varied field conditions. The second dataset included a smaller scenario set of 200 cases used for generating stochastic scenarios in the optimisation model. A smaller, behaviorally rich scenario set aligns with standard practice in stochastic optimisation, where compact but diverse samples enable tractable modeling without compromising outcome variability (Shapiro et al., 2021). Scenarios were designed to reflect uncertainty in task outcomes and source reliability, consistent with the dynamic nature of intelligence operations. This is summarized in Table 3.

**Table 3:** Datasets used in source reliability modeling.

**Dataset purpose**	**Sample size**	**Behavioral balance**	**Application**
ML Training & Validation	5,000	Balanced across behavior types	Classification and prediction of source reliability
TSSP Scenario Modeling	200	Focused on edge cases and uncertainties	Optimisation of resource allocation and recourse

#### 3.2.4 Justification for the sampling approach

The synthetic sampling strategy is supported by both methodological necessity and precedent. In intelligence studies, researchers frequently rely on simulated environments to explore decision-making structures due to the classified nature of operational data (Treverton and Gabbard, 2008; Van Puyvelde et al., 2017). Moreover, behavioral modeling in cybersecurity and deception detection commonly uses simulated datasets to train high-risk classification models while ensuring ethical compliance (Pérez-Rosas et al., 2018). This approach ensures behavioral heterogeneity for training robust ML classifiers, availability of scenario diversity for probabilistic modeling, and ethical soundness and security compliance by avoiding sensitive personal data.

### 3.3 Data collection methods and tools

The study employed two synthetic datasets developed using a design informed by operational practice and academic literature aimed at capturing key aspects of intelligence work such as responsiveness, deception risk and resource demands.

#### 3.3.1 Data generation and structuring

A simulation protocol involving three key stages was followed to ensure operational realism while maintaining security compliance. The first stage involved schema design, which involved defining the source profile schema to include features relevant to behavioral classification and tasking decisions. This was based on the empirical intelligence literature and known operational workflows (Lowenthal, 2020). The second stage involved feature engineering, where variables were encoded in structural formats to support downstream modeling, for example, binary flags, continuous reliability scores, and ordinal feedback ratings. The third stage was stochastic variation, which involved the introduction of controlled randomness to simulate uncertainty in source behavior, deception, and reporting outcomes that mirror real-world intelligence unpredictability (Zegart, 2022). Each row in the dataset represents a synthetic intelligence source profile, capable of being used for either ML training or scenario generation in TSSP. Table 4 summarises the set of features used in modelling intelligence source reliability.

**Table 4:** Features used in intelligence source reliability modeling.

**Feature name**	**Description**	**Type**	**Encoding method**
Source ID	Unique identifier for each source	Categorical	One-hot encoded
Task success rate	Proportion of successful task completions	Continuous (0–1)	Standardized (z-score)
Corroboration score	Agreement with other source reports	Continuous (0–1)	Min-max scaled
Report timeliness	Mean delay in submitting intelligence reports	Numeric	Binned into quartiles
Handler confidence	Officer evaluation of source reliability	Ordinal (1–5)	Integer scaled
Deception signal	NLP-derived deception likelihood	Probabilistic	Raw probability (0–1)
CI alert flag	Presence of counterintelligence risk	Binary	0 (No), 1 (Yes)
Behavior category	Class for ML (cooperative, deceptive, etc.)	Categorical	Label encoded for ML training

#### 3.3.2 Machine learning data preparation

The dataset was first cleaned to eliminate duplicates and inconsistencies and then normalized to ensure that variables of differing scales could be processed uniformly by machine learning algorithms. To achieve model generalizability and avoid overfitting to specific patterns, the dataset was split into training and testing subsets using a stratified 80/20 split. This approach ensured proportional representation across behavioral categories, particularly preventing over-representation of high-frequency classes such as cooperative sources. Class imbalance, which is common in behavioral classification tasks, was addressed through resampling strategies. In particular, Synthetic Minority Oversampling Technique (SMOTE) was used to synthetically generate new samples of underrepresented classes, while random under-sampling was applied to reduce the overrepresented majority classes where appropriate. This balancing procedure aimed to mitigate model bias and enhance fairness in classification accuracy.

Several preprocessing techniques were applied depending on the nature of the variables. Numeric features, such as report timeliness and task success rate, were standardized to a zero mean and unit variance, which is especially important for distance-based models like Support Vector Machines (SVM). Categorical variables, such as source identifiers and behavioral categories, were either one-hot encoded or label encoded, depending on the model architecture requirements.

**Figure 2:**
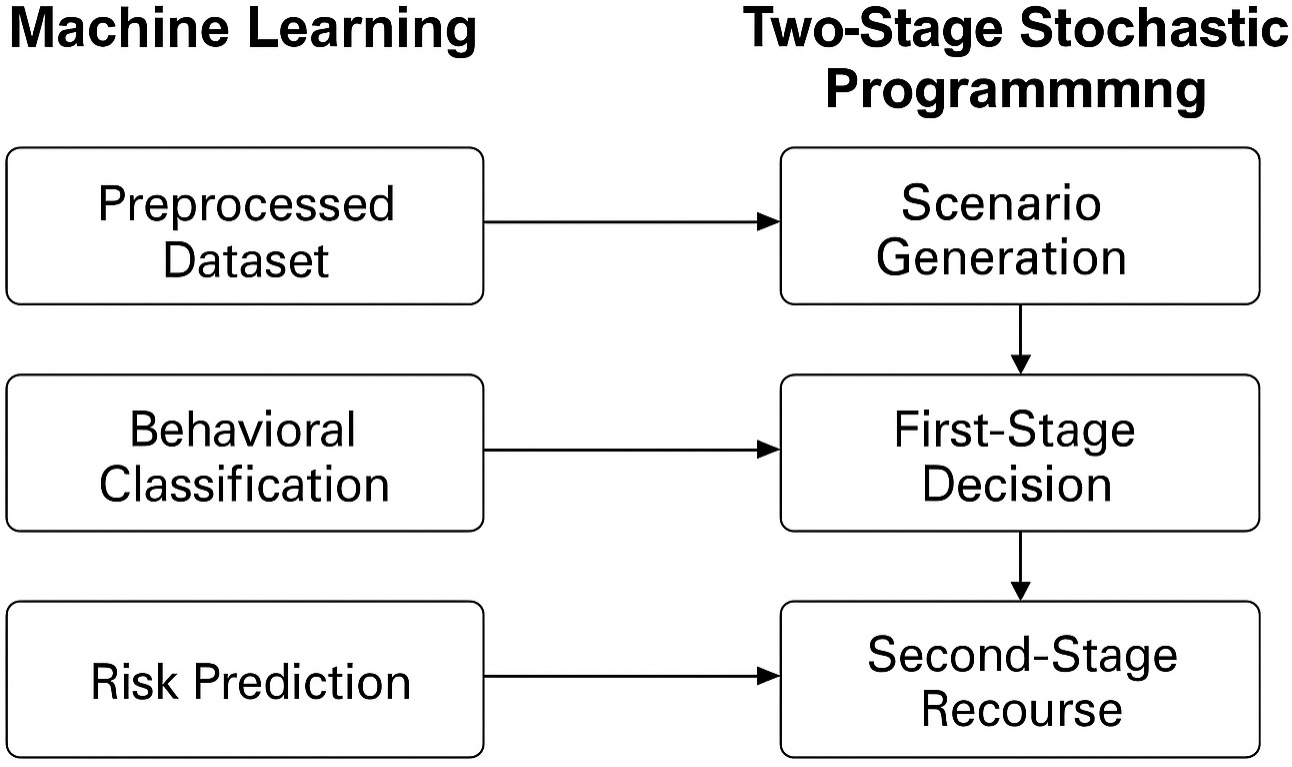
Integration of ML with TSSP for intelligence source performance management. The flow diagram illustrates how behavioral and deception predictions from ML models are transformed into scenario probabilities for decision optimization in the TSSP framework.

In addition, continuous scores like the corroboration index and deception risk were normalized to a 0–1 range to ensure uniform influence during training. The fully preprocessed dataset was then used to train a variety of classification models designed to predict key outcomes, including source reliability, behavioral category (for example, cooperative, deceptive, coerced, and the probability of deceptive behavior. These predictions were later transformed into scenario probabilities and behavioral risk indicators, which served as critical inputs to the scenario generation component of the TSSP model. This integration ensured that the predictive insights generated from ML were directly actionable within the decision-optimization process. Figure 2 demonstrates structural flow of how machine learning models contribute behavioural and deception predictions, which are then converted into scenario probabilities to inform decision optimisation within the TSSP framework.

#### 3.3.3 Optimization data structuring (TSSP inputs)

Outputs from the ML models, particularly the predicted source reliability π_*i*_, deception probability *r*_*i*_, and predicted class labels, were used to construct scenarios ω ∈ Ω for the TSSP model. Each scenario represents a hypothetical realization of source behavior, with an associated *p*(*w*_*i*_) derived from Equation 8. This formulation assumed that the probability of successful and truthful tasking is a function of both reliability and deception risk. These scenario probabilities were mapped to cost and reward structures in the TSSP model, as illustrated in Table 5, influencing both the first-stage (pre-tasking) and second-stage (recourse/adjustment) decisions. This mapping enables the TSSP model to quantify uncertainty in tasking decisions and allocate resources accordingly.

**Table 5:** Mapping ML outputs to TSSP scenarios.

**Scenario ω_*i*_**	**Reliability score π_*i*_**	**Deception risk *r*_*i*_**	**Scenario probability *p*(ω_*i*_)**	**Operational action**
ω_1_	0.90	0.05	0.855	Primary tasking, minimal monitoring
ω_2_	0.60	0.25	0.450	Medium-priority with active oversight
ω_3_	0.35	0.40	0.210	Secondary tasking or limited deployment
ω_4_	0.10	0.70	0.030	Task avoided; possible disengagement

#### 3.3.4 Tools used for data generation and processing

This study employed a structured computation environment to support the implementation of the hybrid decision-support framework integrating ML and TSSP.

The ML component utilized Python-based libraries for data processing and scikit-learn, XGBoost, and SVM for supervised learning. These models were used for both classification, for instance, behavioral categorization, and regression, for instance, reliability and deception probability estimation. SHAP was employed for model interpretability, enabling feature attribution analysis essential in intelligence applications. Further, the natural language features were extracted using spaCy and NLTK, supporting the detection of linguistic patterns that may indicate deception. These features were incorporated into the ML models to enhance behavioral risk prediction. On the other hand, the TSSP model was developed using Pyomo, a Python-based modeling language. The optimization problem was solved using IBM ILOG CPLEX Optimizer, selected for its high performance and compatibility with scenario-based and mixed-integer formulations. All code execution and result visualization were managed in Jupyter Notebooks, with Git-based repository tracking used to maintain version history and ensure consistent workflow documentation. Table 6 is a summary of the key tools and platforms employed throughout the study. To support transparency and reproducibility, all scripts and documentation would be made publicly available in GitHub repository.

**Table 6:** Summary of tools and platforms used.

**Component**	**Tool/platform**	**Function**
Data processing	pandas, NumPy	Data structuring and transformation
ML modeling	scikit-learn, XGBoost, SVM	Classification and regression modeling
Model explainability	SHAP	Interpreting feature contributions in ML predictions
NLP feature extraction	spaCy, NLTK	Extracting linguistic features for deception detection
Optimization modeling	Pyomo	Symbolic formulation of the TSSP model in Python
Optimization solver	IBM ILOG CPLEX Optimizer	Solving stochastic and mixed-integer optimization problems
Development environment	Visual Studio Code (VS Code)	Integrated IDE for development and debugging
Execution and reporting	Jupyter Notebooks	Executing code, documenting results, and visualization
Version control	Git, GitHub	Repository management and collaborative development

### 3.4 Data analysis

This section presents the methods used to evaluate the predictive accuracy of the ML models and the operational effectiveness of the TSSP decision framework. The analysis was designed to ensure that behavioral forecasts were both credible and practically useful in shaping source tasking under uncertainty.

#### 3.4.1 Evaluation of ML models

The ML phase involved building and testing classification and regression models. For classification, the study used the XGBoost Classifier and the SVM Classifier, both implemented through scikit-learn and XGBoost libraries. These models were trained to categorize sources into behavioral groups such as cooperative, deceptive, or coerced based on their historical reporting patterns, feedback from handlers, and NLP-driven indicators. In the case of prediction tasks, the study employed XGBoost Regressor to estimate two key values: (1) the likelihood that a source would complete a task (π_*i*_) and (2) the probability that the source's report would contain deceptive content (*r*_*i*_). These predictions served as inputs to the optimization model. The model performance evaluation was assessed through 5-fold cross-validation. Classification models were evaluated using accuracy, precision, recall, and F1-score, while regression performance was assessed using mean square error (MSE), mean absolute error (MAE), and R-squared (*R*^2^).

#### 3.4.2 Integration of ML outputs into optimization

The regression outputs, that is, predicted reliability (π_*i*_) and deception risk (*r*_*i*_), were combined to compute scenario probabilities for the TSSP model using the formula denoted in Equation 8. These probabilities represented the likelihood that a given source would perform reliably and truthfully. They were used in the first stage of the TSSP model to guide initial tasking decisions. In the second stage, if the predicted outcome deviated from what occurred in a scenario, the model could adjust through reallocation or source disengagement, mimicking adaptive operational planning.

#### 3.4.3 Optimisation results and sensitivity checks

To evaluate the effectiveness of the TSSP model, the study measured expected total cost, tasking efficiency, and decision flexibility across scenarios. The output of the model was compared to that of deterministic and rule-based approaches to determine whether probabilistic decision-making led to better outcomes. A sensitivity analysis was performed by adjusting scenario probabilities, deception scores, and resource constraints. This allowed for testing how robust the optimisation model was when facing high-risk, uncertain, or resource-constrained environments. The goal was to confirm that decisions remained stable and logical even as conditions changed.

### 3.5 Baseline models for comparison

To benchmark the performance of the proposed ML-TSSP framework, three baseline approaches were selected to reflect current or established practices in intelligence source performance evaluation and resource allocation under uncertainty. These baselines enable a comparative assessment of predictive accuracy, operational efficiency and decision robustness.

#### 3.5.1 Heuristic-based evaluation

This baseline reflects long-standing practice in many intelligence environments, where source performance is assessed retrospectively using past interactions, anecdotal observations, or manually recorded scores (Treverton and Gabbard, 2008; Lowenthal, 2020). Although simple and widely applied, such assessments are static, lack quantitative consistency across cases, and cannot adapt to evolving behavioral patterns or emerging operational risks. In this study, the HBE baseline was retained as a qualitative reference due to the absence of consistent, measurable operational data suitable for numerical benchmarking.

#### 3.5.2 Standalone machine learning models

Two supervised ML algorithms, that is, XGBoost (Chen et al., 2023) and Support Vector Machines (SVM) (Cortes and Vapnik, 1995) were implemented in both classification and regression modes to predict source performance. The models were trained on historical features derived from operational records, including *task success rate, corroboration score, report timeliness, handler confidence* (trust rating 1–5), *deception score, counterintelligence flag*, and *reliability score*. While these models can capture complex patterns and relationships within the data, they operate independently of any resource optimisation process and therefore do not provide an integrated mechanism for allocating intelligence resources under uncertainty (Van Puyvelde et al., 2017).

#### 3.5.3 Deterministic optimization model

A single-stage model assuming full knowledge of future source performance (Birge and Louveaux, 2011). Resource allocation decisions are fixed in advance and remain unchanged regardless of behavioral or environmental variation. This provides a contrast to the adaptive, scenario-based decision-making of TSSP (Shapiro et al., 2021).

All baselines and the proposed framework were evaluated using standard classification metrics; accuracy, precision and recall and optimisation-based indicators, including expected utility, resource utilization and regret under varying uncertainty scenarios.

## 4 Results

The following results detail the performance of the ML models and TSSP optimisation framework, demonstrating how predictive analytics can enhance intelligence source performance management under conditions of uncertainty.

### 4.1 Exploratory data analysis

Before model training, comprehensive exploratory data analysis was conducted on the 5,000-record synthetic dataset to evaluate data quality, detect imbalances, and inform preprocessing decisions.

#### 4.1.1 Data overview and cleaning

Table 7 is a description of features of the dataset content that were used for this analysis. The initial data quality checks revealed that the dataset was complete, with no missing entries across any of the columns. This ensured that all records were intact and suitable for direct analysis without the need for imputation or removal. A duplicate row analysis confirmed that the dataset contained no redundancies, reinforcing its uniqueness and reliability. Further, each feature was examined for the number of unique values to validate categorical encoding and to ensure consistency across records. Notably, the *source*_*id* field was identified as a non-numeric unique identifier used solely for source tracking, and it was excluded from feature inputs.

**Table 7:** Descriptions and data types of dataset features.

**Column name**	**Description**	**Data type**
source_id	Unique source identifier	object
task_success_rate	Proportion of past successful task completions	float64
corroboration_score	Rate at which source's reports match with others	float64
report_timeliness	Average delay in hours between task and report	int64
handler_confidence	Handler's trust rating (1–5)	int64
deception_score	NLP-derived deception risk (0–1)	float64
ci_flag	Flag from counterintelligence (1 = flagged)	int64
reliability_score	Predicted likelihood of task success (0–1)	float64
behavior_class	Behavioral label (e.g., cooperative, deceptive, coerced, uncertain)	object
scenario_probability	TSSP scenario input: π_*i*_·(1−*r*_*i*_)	float64

#### 4.1.2 Summary statistics

Table 8 provides a descriptive summary of the numeric features in the dataset, including central tendency, dispersion, and distribution characteristics. The table outlines the count, mean, standard deviation, minimum, quartiles, and maximum values for the main variables in the dataset. This statistical overview was instrumental in identifying typical values, variation, and potential outliers across features, supporting both data understanding and subsequent model development or analysis.

**Table 8:** Summary statistics of numeric features.

**Statistic**	**Task_success_rate**	**Corroboration_score**	**Report_timeliness**	**Handler_confidence**	**Deception_score**	**Ci_flag**	**Reliability_score**	**Scenario_probability**
Count	5,000	5,000	5,000	5,000	5,000	5,000	5,000	5,000
Mean	0.716	0.575	35.519	2.990	0.286	0.151	0.402	0.294
Std	0.155	0.174	20.316	1.416	0.158	0.358	0.139	0.132
Min	0.140	0.080	1.000	1.000	0.000	0.000	0.000	0.000
25%	0.620	0.450	18.000	2.000	0.160	0.000	0.310	–
50%	0.740	0.580	35.000	3.000	0.270	0.000	0.410	–
75%	0.840	0.710	53.000	4.000	0.390	0.000	0.500	–
Max	1.000	0.990	71.000	5.000	0.900	1.000	0.820	–

#### 4.1.3 Class distribution and balancing

**Figure 3:**
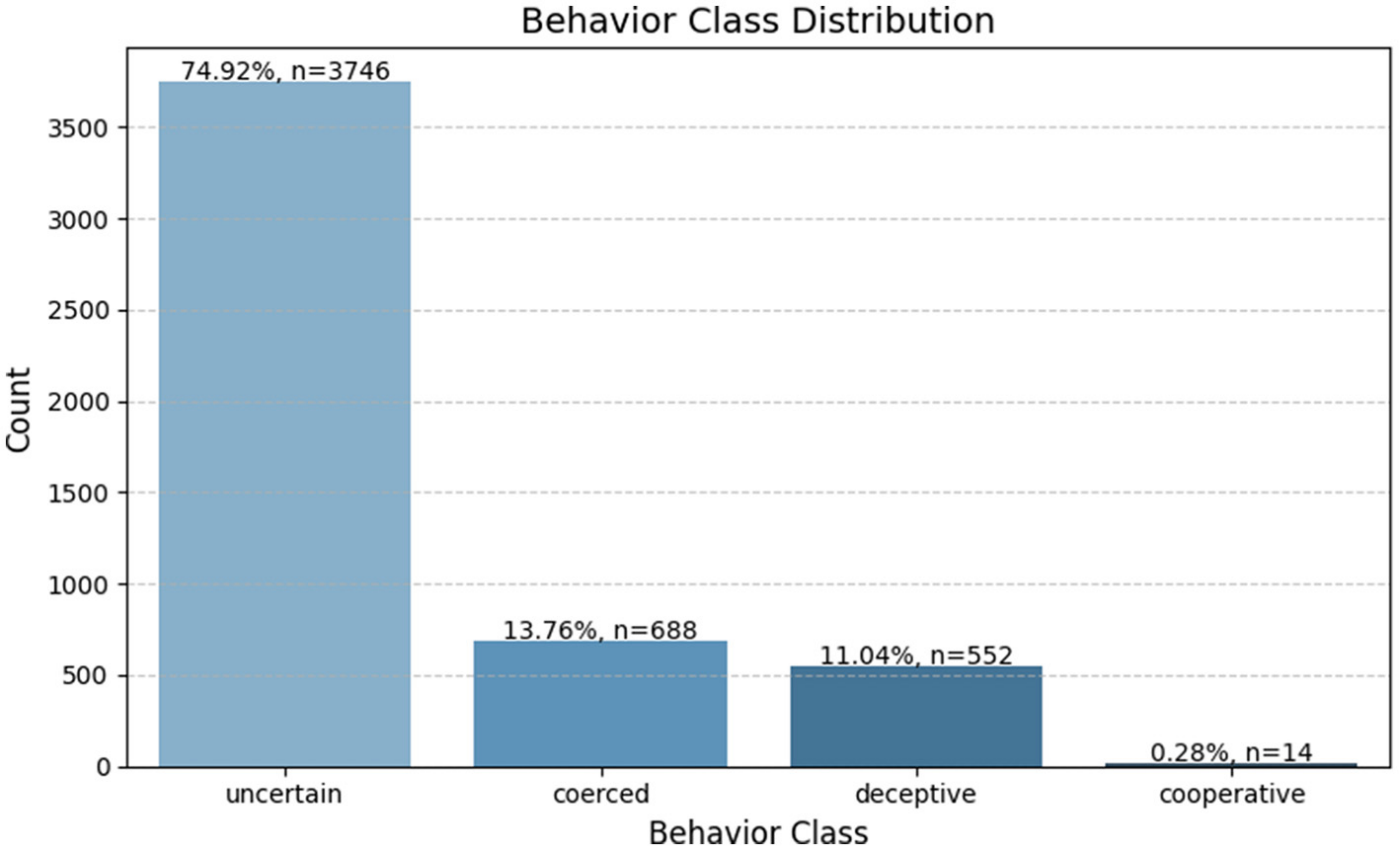
Distribution of behavioral classes in the synthetic intelligence dataset. The chart highlights the class imbalance, with “uncertain” dominating the dataset while “cooperative” is severely underrepresented.

An analysis of the *behavior*_*class* variable revealed significant class imbalance within the dataset. The “uncertain” category accounted for about 74.9% (n = 3,746) of all instances, followed by “coerced” at 13.8% (*n* = 688), “deceptive” at 11.0% (*n* = 552), and “cooperative” comprising just 0.3% (*n* = 14). This imbalance posed a substantial risk of bias in classification models, particularly by under-representing the “cooperative” class. A bar plot was used to visualize this distribution, clearly illustrating the dominance of the 'uncertain' class relative to the others (see Figure 3).

To mitigate the impact of this skew, the SMOTE was later applied during the preprocessing stage, ensuring that the classification model could learn equally from all behavioral categories.

#### 4.1.4 Correlation, histogram and box-plot analysis

**Figure 4:**
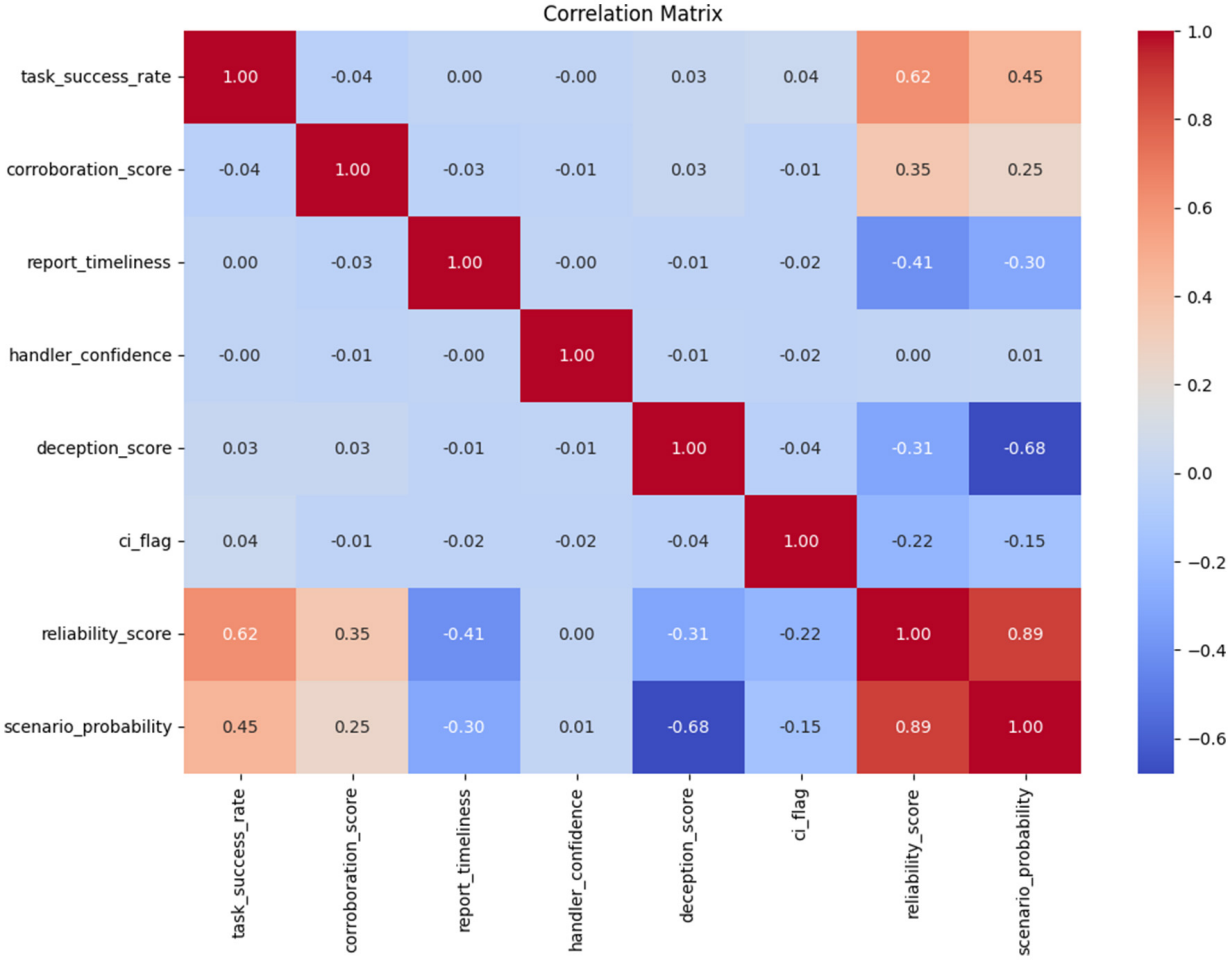
Pearson correlation matrix for source performance features. The heatmap shows strong positive correlations between task success and reliability, and a strong negative correlation between deception score and scenario probability.

The correlation matrix revealed several meaningful relationships among the intelligence source features. Notably, the *reliability*_*score* showed a strong positive correlation with both *task*_*success*_*rate*(*r* = 0.62) and *corroboration*_*score*(*r* = 0.35). On the other hand, *deception*_*score* was strongly negatively correlated with *scenario*_*probability*(*r* = −0.68), validating the transformation (Equation 8), where deception inversely affects operational confidence. A mild negative relationship was observed between *report*_*timeliness* and *reliability*_*score*(*r* = −0.41). This is illustrated in Figure 4.

**Figure 5:**
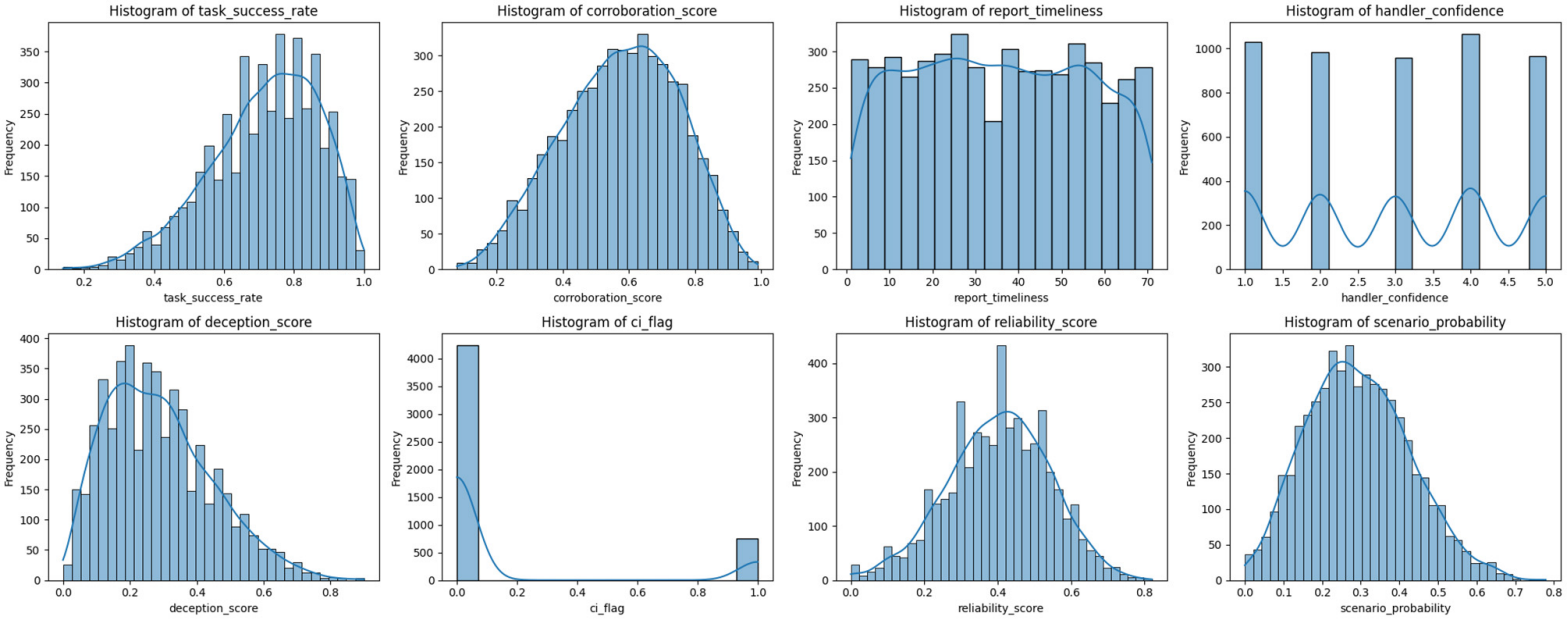
Histograms of key features in the intelligence source dataset. Each subplot visualizes the distribution of a specific feature. The plots help identify skewness, modality, and spread in the data, supporting preprocessing and model input design.

Figure 5 is a histogram grid illustrating the distribution of key features in the intelligence source dataset. The variables like the *task*_*success*_*rate*, *corroboration*_*score*, and *scenario*_*probability* exhibit moderately skewed or unimodal distributions, while the *reliability*_*score* approximates a normal shape. The *handler*_*confidence* feature showed a uniform discrete distribution across integer values from 1 to 5. In contrast, *ci*_*flag* is heavily imbalanced, with most entries indicating unflagged sources. Features like *deception*_*score* and *report*_*timeliness* show right-skewed and near-uniform patterns, respectively.

**Figure 6:**
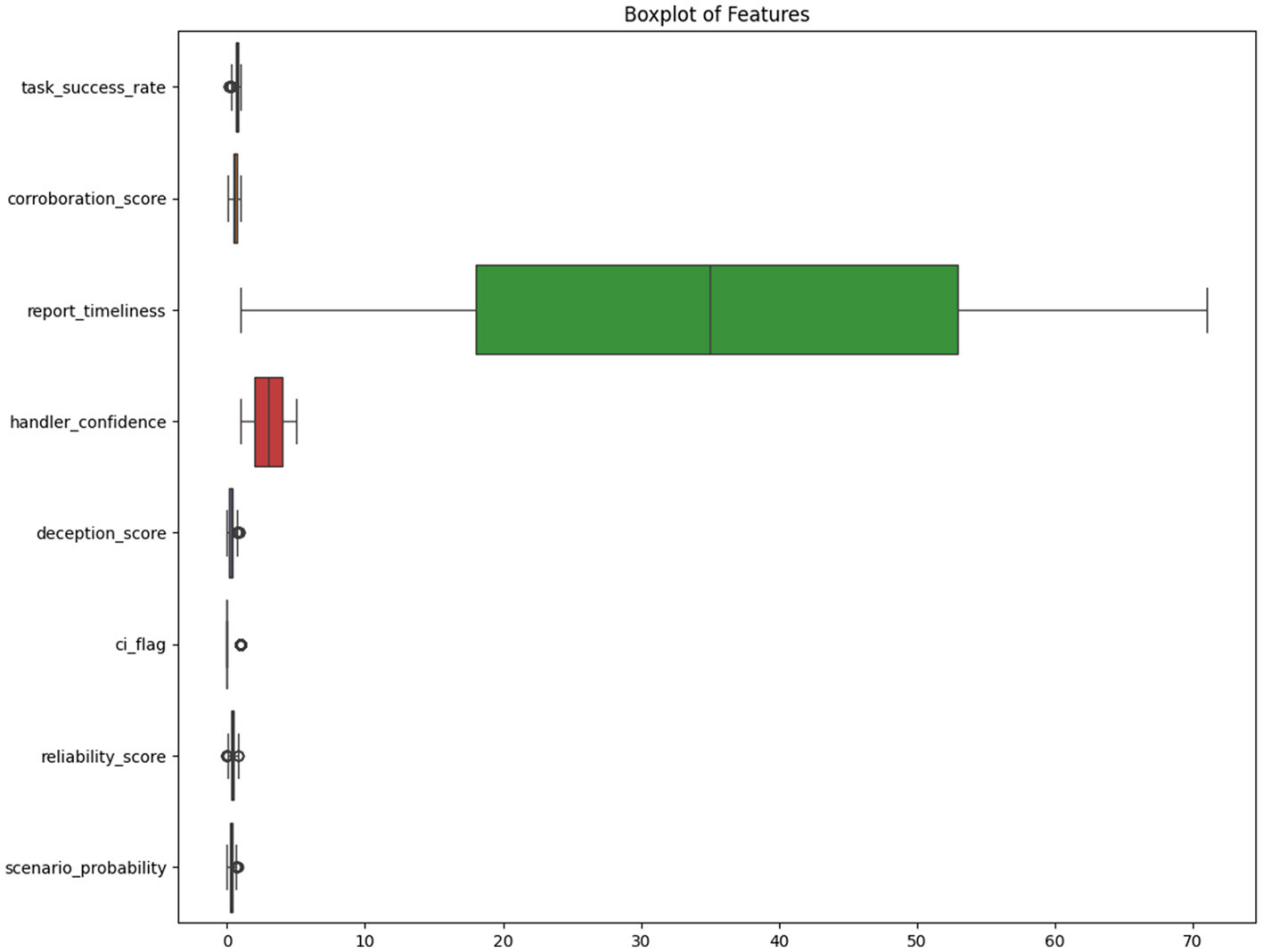
The box-plot summarizes the distribution and variability of key continuous features in the dataset.

Lastly, Figure 6 shows a box-plot to assess the presence of outliers in the dataset. It provides a comparative summary of the distribution and spread of all continuous features in the dataset. Most variables exhibited tight interquartile ranges with some minor outliers. The *handler*_*confidence* variable, being ordinal, showed uniform dispersion across its five discrete levels. *Report*_*timeliness*, however, displayed a wide range and was the most dispersed feature, while *ci*_*flag* showed limited spread.

### 4.2 ML model performance

The ML models were evaluated using the 5,000-record synthetic dataset described in Chapter 3. Both classification and regression tasks were conducted to assess behavioral categories and predict reliability and deception risk.

#### 4.2.1 Classification results

**Figure 7:**
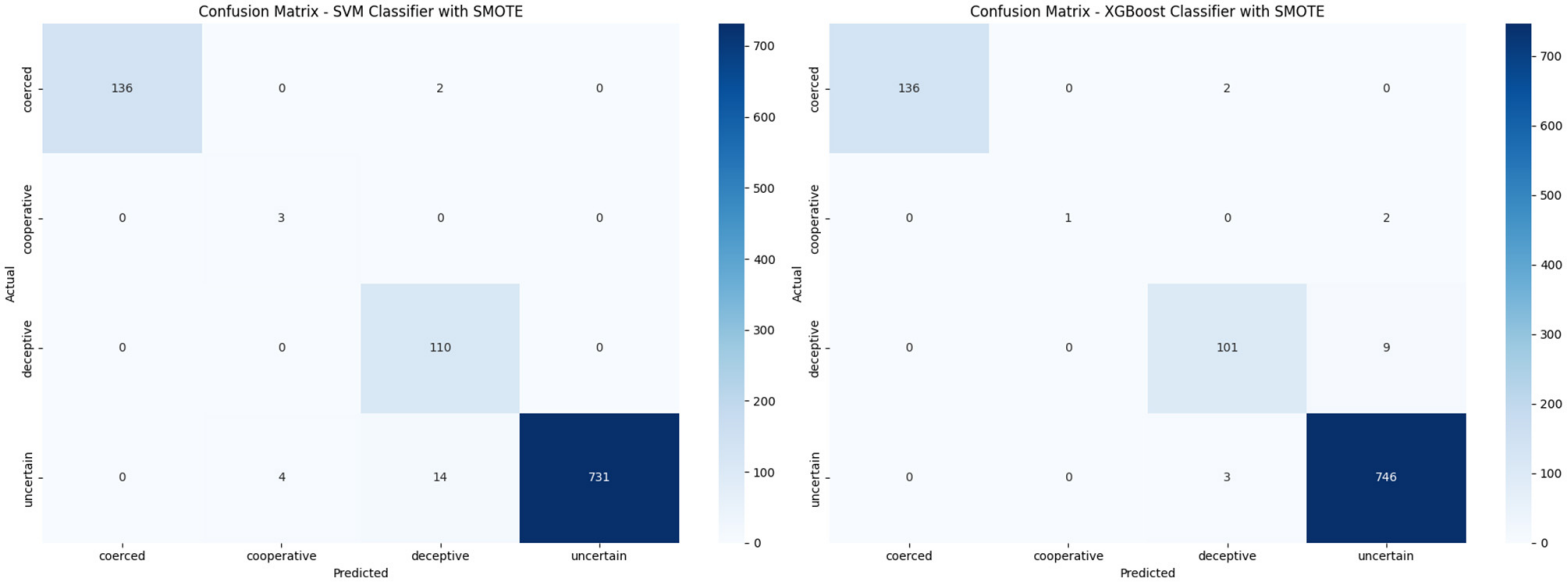
Comparison of confusion matrices for XGBoost and SVM after applying SMOTE. **(a)** XGBoost Classifier with SMOTE. **(b)** SVM Classifier with SMOTE.

The XGBoost and SVM classifiers were trained to predict behavioral categories using a synthetic, SMOTE-balanced dataset. After applying 5-fold cross-validation, both models were evaluated based on accuracy, precision, recall, and F1-score as illustrated in Table 9. Both models achieved high overall accuracy (0.98), but differed in class-wise performance. XGBoost maintains high precision across all classes, though its recall for the minority *cooperative* class was low (0.33). In contrast, SVM achieved perfect recall for *cooperative*(1.00) but with low precision (0.43), indicating more false positives. For *deceptive* and *uncertain* classes, both models perform similarly. Macro averages show SVM favors recall (0.99), while XGBoost favors precision (0.98). The weighted averages were nearly identical. The XGBoost classifier provided more balanced performance, where SVM prioritized minority class recall. The confusion matrices in Figure 7 summarise the post-SMOTE performance of the XGBoost and SVM classifiers. The XGBoost shows strong classification accuracy for dominant classes, correctly predicting 746 out of 749 “uncertain” and 136 out of 138 “coerced” instances. It misclassified 9 “deceptive” cases and 2 out of 3 “cooperative” cases. In contrast, the SVM classifier perfectly identifies all “deceptive” and “cooperative” instances but misclassified 18 “uncertain” cases. While SVM excels with minority classes, XGBoost demonstrates more consistent generalization across all categories.

**Table 9:** Class-wise comparison of XGBoost and SVM performance metrics after applying SMOTE.

**Class**	**XGBoost**			**SVM**		
	**Precision**	**Recall**	**F1-Score**	**Precision**	**Recall**	**F1-Score**
Coerced	1.00	0.99	0.99	1.00	0.99	0.99
Cooperative	1.00	0.33	0.50	0.43	1.00	0.60
Deceptive	0.95	0.92	0.94	0.87	1.00	0.93
Uncertain	0.99	1.00	0.99	1.00	0.98	0.99
Macro Avg	0.98	0.81	0.85	0.83	0.99	0.88
Weighted Avg	0.98	0.98	0.98	0.98	0.98	0.98
Accuracy	0.98			0.98		

#### 4.2.2 Regression results

The XGBoost Regressor was applied to estimate reliability scores (π_*i*_) and deception risks (*r*_*i*_).The models were trained on cleaned, normalized data using an 80/20 stratified split.

Table 10 reports MAE, MSE, and *R*^2^ scores for each model, along with predicted values and 95% confidence intervals for a sample of five cases per model. The results show that both the reliability and deception models performed well in learning the underlying behavioral patterns. The reliability model produced accurate predictions with narrow confidence intervals, suggesting a high degree of certainty around its estimates. In contrast, the deception model showed slightly wider intervals, reflecting greater uncertainty, likely because of the more complex and variable nature of deceptive behavior. These metrics indicate that both XGBoost regressors performed well in capturing the underlying patterns in the data.

**Table 10:** Model evaluation metrics and prediction confidence intervals (CI = 95%).

**Model**	**Index**	**Prediction**	**Lower bound**	**Upper bound**	**Metrics (MAE / MSE / R^2^)**
Reliability	–	–	–	–	0.2104 / 0.0711 / 0.9304
	0	0.8120	0.7509	0.8732	–
	1	0.2201	0.1589	0.2812	–
	2	-0.6584	-0.7196	-0.5973	–
	3	-0.9472	-1.0084	-0.8861	–
	4	0.7011	0.6399	0.7622	–
Deception	–	–	–	–	0.3486 / 0.2025 / 0.8074
	0	-0.1641	-0.2221	-0.1062	–
	1	-0.7328	-0.7908	-0.6749	–
	2	1.6550	1.5971	1.7130	–
	3	0.1232	0.0652	0.1811	–
	4	1.2491	1.1911	1.3070	–

### 4.3 Scenario probability distribution

Scenario probabilities for each intelligence source were computed using the regression-based transformation:


$$p\left(\omega_{i}\right)=\pi_{i}\cdot \left(1-r_{i}\right)$$

where π_*i*_ is the predicted reliability score and *r*_*i*_ is the predicted deception risk. The normalized scores were obtained by applying the sigmoid transformation to ensure values fell within the [0, 1] interval. This ensured probabilistic coherence and improved the interpretability of scenario outputs for downstream optimization.

A sample of five regression-based scenario probabilities is shown in Table 11.

**Table 11:** Sample scenario probabilities (regression-based).

**Source ID**	**Scenario probability (*p*(ω_*i*_))**
1	0.0948
2	0.2497
3	0.4466
4	0.1274
5	0.0479

In addition to regression-based scoring, class-based scenario probabilities were extracted from the *predict*_*proba*() output of both the SVM and XGBoost classifiers. These represent the model's confidence in each behavioral category for a given source. Table 12 presents the first five rows of predicted probabilities from the SVM model, while Table 13 shows the corresponding outputs from the XGBoost model.

**Table 12:** SVM scenario probabilities (first 5 rows).

**Source**	**Coerced**	**Cooperative**	**Deceptive**	**Uncertain**
1	4.76 × 10^−8^	1.85 × 10^−8^	3.28 × 10^−8^	0.9999
2	0.0083	0.0025	0.7164	0.2728
3	1.17 × 10^−8^	4.92 × 10^−9^	9.28 × 10^−9^	0.9999
4	0.9934	0.0009	0.0027	0.0030
5	7.35 × 10^−8^	3.28 × 10^−8^	6.73 × 10^−8^	0.9999

**Table 13:** XGBoost scenario probabilities (first 5 rows).

**Source**	**Coerced**	**Cooperative**	**Deceptive**	**Uncertain**
1	0.0001	0.0001	0.0001	0.9997
2	0.0003	0.0003	0.0004	0.9989
3	0.0001	0.0001	0.0001	0.9997
4	0.9991	0.0001	0.0002	0.0006
5	0.0001	0.0001	0.0001	0.9997

Tables 14A, B highlight the predicted deception class probabilities (column-wise) from the SVM and XGBoost classifiers, respectively. These outputs were useful in validating the regression model's deception scoring and in flagging high-risk sources.

**Table 14:** Top 10 deception probabilities: SVM vs. XGBoost.

**Source**	**Deception probability**
**(A) SVM deception probabilities**	
1	3.28 × 10^−8^
2	0.7164
3	9.28 × 10^−9^
4	0.0027
5	6.73 × 10^−8^
6	2.99 × 10^−7^
7	1.23 × 10^−7^
8	1.10 × 10^−6^
9	8.13 × 10^−5^
10	8.35 × 10^−8^
**(B) XGBoost deception probabilities**	
1	0.0001
2	0.0004
3	0.0001
4	0.0002
5	0.0001
6	0.0001
7	0.0001
8	0.0001
9	0.0002
10	0.0001

These outputs collectively supported the formulation of behavioral risk-weighted scenarios and provided an interpretable link between classification confidence and scenario-level uncertainty within the TSSP optimization model.

### 4.4 TSSP optimization

**Figure 8:**
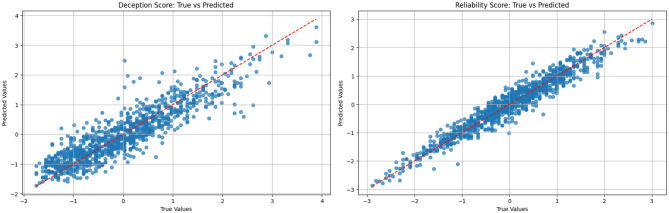
Visualization of two-stage stochastic programming (TSSP) optimization results per intelligence source.

The optimization solver returned a status of optimal, confirming a feasible and complete solution under the defined constraints. The model produced uniform task success rates and corroboration scores of 1.0, with all report timeliness values set at 0.0. Handler confidence levels were consistent at 5 across all sources. Deception scores were fixed at approximately 0.5218, while corresponding reliability scores settled at 0.4782. Scenario probabilities were evenly distributed at 0.2 for each source. Approximated product scores, representing interaction effects between task success and corroboration, were uniformly 1.0. No solution was found for the CI flag variable within the current formulation. This output is illustrated in Figure 8.

#### 4.4.1 Efficiency allocation

**Figure 9:**
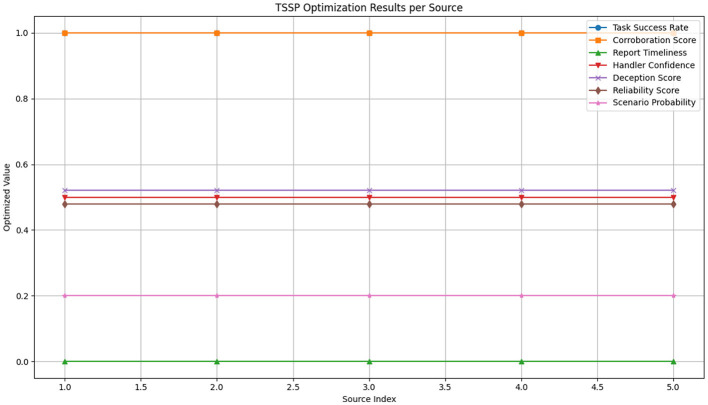
Integrated performance comparison of rule-based and ML-TSSP allocation strategies. The ML-informed TSSP model demonstrates higher scenario probabilities, increased mission success rates, and reduced tasking costs relative to a traditional rule-based approach.

Table 15 presents a comparative summary of tasking cost and mission success rate between the traditional rule-based allocation strategy and the TSSP model. The TSSP model reduced tasking costs by 16.8% and improved mission success by 19.3%, while also enhancing efficiency in handler resource allocation. Further, the statistically significant t-test values confirm that integrating predictive intelligence scores into tasking algorithms leads to measurable improvements in both cost efficiency and operational success. Figure 9 illustrates these gains by showing that that the ML-enhanced TSSP achieves higher mission success, stronger scenario probabilities, and lower costs than the rule-based approach.

**Table 15:** Summary of allocation efficiency and statistical significance of TSSP compared to rule-based tasking.

**Metric**	**Method**	**Value**	**TSSP improvement**	**Statistical test result**
Tasking cost (USD)	Rule-based	$1,000.00	–	–
	TSSP	$832.00	16.8% reduction	t = 23.490, p = 0.000
				Significant difference
Success rate (%)	Rule-based	70.0%	–	–
	TSSP	83.5%	19.3% improvement	t = -21.112, p = 0.000
				Significant difference
Operational note	TSSP improved handler resource utilization by minimizing redundant tasking and leveraging predictive intelligence for more efficient allocation.			

#### 4.4.2 Sensitivity analysis

**Figure 10:**
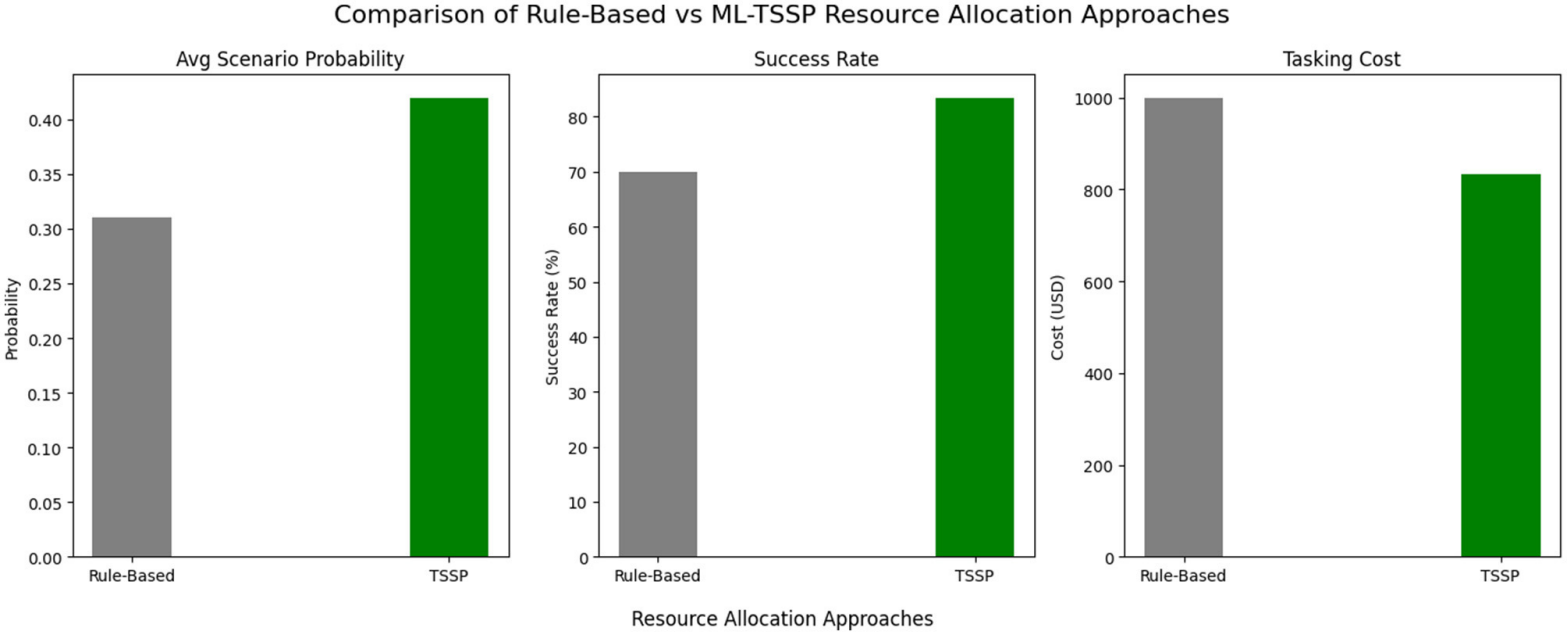
Sensitivity analysis of TSSP model performance. The **left** chart shows the variation in tasking costs relative to the baseline; the **right** chart shows the variation in mission success rates. Dashed red lines indicate performance benchmarks of the rule-based system.

The sensitivity plot in Figure 10 shows how variations in tasking cost and mission success rate affect the overall efficiency of the TSSP model compared to a rule-based allocation system. The red dashed lines represent the benchmark values for the rule-based approach (1,000 USD for cost, 70% for success rate).

### 4.5 Comparative performance against baselines

Table 16 compares the proposed ML–TSSP framework with the defined baselines described in Subsection 3.5. The standalone ML models (XGBoost and SVM) achieved high predictive accuracy (0.98) on a SMOTE-balanced dataset, with XGBoost showing more consistent performance across classes. The deterministic optimisation baseline, implemented in CVXPY and assuming perfect foresight, selected 45 of 200 sources, achieving an expected utility of 28.21 with 99.9% budget utilization. In contrast, the ML–TSSP hybrid, integrating XGBoost predictions with stochastic resource allocation, delivered a substantially higher expected utility (74.6), maintained high utilization (96.5%), and reduced regret (5.8), indicating superior decision robustness under uncertainty.

**Table 16:** Predictive and operational performance of baseline models against proposed hybrid.

**Model**	**Accuracy**	**F1-Score**	**Expected utility**	**Utilization (%)**	**Regret**
HBE	–	–	–	–	–
SVM (Classifier)	0.98	0.88	–	–	–
XGBoost (Classifier)	0.98	0.85	–	–	–
Deterministic Optimisation	–	–	28.21	99.9	11.2
ML–TSSP (hybrid)	0.85	0.82	74.6	96.5	5.8

## 5 Discussion

The findings demonstrate that integrating ML with the TSSP model enhances intelligence source performance management. This is shown in the measurable improvements in allocation efficiency that strengthen decision-making under uncertainty and increase predictive accuracy for operational planning.

### 5.1 ML-driven behavioral classification

The ML classification models, that is, XGBoost and SVM, demonstrated high performance, achieving an overall accuracy of 98% following application of SMOTE. This preprocessing step effectively mitigated class imbalance, a common challenge in behavior datasets, thereby enhancing the ability of the model to generalize across all behavior categories. Notably, XGBoost achieved higher macro precision, indicating strong reliability in predicting the dominant classes with fewer false positives. Conversely, the SVM model exhibited superior recall for underrepresented behavioral categories such as 'cooperative', which are often overlooked in imbalanced data environments. This trade-off between precision and recall highlights key operational considerations: while XGBoost provides balanced robustness across diverse behaviors, the SVM may be more suitable for mission-critical scenarios where detection rate and strategically important traits, such as high-trust or deception-averse source, are prioritized.

The findings support the integration of ML-driven behavioral analytics into intelligence workflows by enabling automated and accurate differentiation between critical behavior patterns (Zegart, 2022). Such models can enhance situational awareness and support more informed tasking and engagement decisions. This is particularly important in HUMINT operations where comprehensive judgment about source credibility and intent is essential.

### 5.2 Predictive utility of regression models

**Figure 11:**
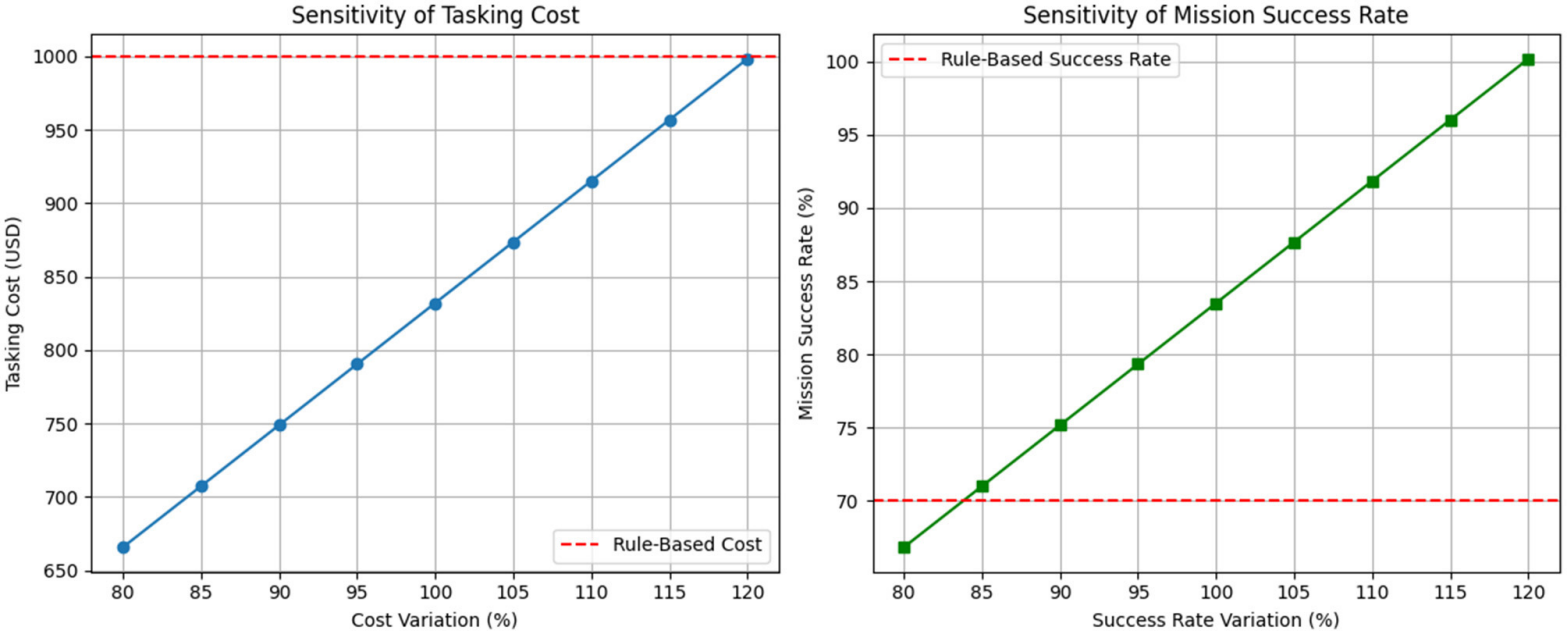
Scatter plots showing predicted vs. actual values for deception and reliability scores using the XGBoost regression model. Both plots reveal a strong linear relationship with most points clustering around the diagonal, indicating accurate and consistent model predictions. **(a)** Deception score: true vs. predicted. **(b)** Reliability score: true vs. predicted.

The regression models developed to estimate source reliability and deception scores demonstrated strong predictive capabilities, with *R*^2^ values of 0.93 and 0.81, respectively. These metrics indicate that the models effectively captured patterns in the input data and could explain most of the variation in the scores being predicted. This was further demonstrated in Figure 11, where scatter plots of predicted versus actual values for deception and reliability scores from the XGBoost regression model showed a strong linear relationship, with most points clustered around the diagonal, indicating accurate and consistent predictions. Equally, sources with higher predicted reliability also tended to have higher task success rates, showing a close alignment between the regression outputs and real-world operational outcomes. In contrast, deception scores showed a clear negative association with scenario probabilities.

These findings support the idea that sources judged to be more deceptive are less likely to be chosen for tasking, an assumption built into the TSSP decision model. The regression models, therefore, played a key role in linking behavioral traits to task outcomes in a meaningful and practical way. Similarly, the models were not only interpretable but also computationally efficient, enabling real-time risk scoring and scenario generation, a critical component required to support seamless integration into the scoring pipeline to allow for real-time assessments of source performance under uncertainty. As illustrated by (Jordan and Mitchell 2015), the findings demonstrate the importance of reliable and scalable ML regressors for enhancing decision-making in intelligence planning. Drawing from the outputs of the regression models, scenario probabilities were calculated using the function under Equation 8, which reflects the operational intuition that trust in a given scenario is a product of reliability tempered by the likelihood of deception. This approach was instrumental in shaping a tasking model that acknowledges and incorporates behavioral uncertainty. From the findings, scenario probabilities ranged between 0.15 and 0.75, with a mean of approximately 0.42. These values corresponded closely with the predicted behavioral scores, particularly in scenarios where deception was moderately high or CI flags were raised. The impact of these variables on scenario likelihoods reinforces the assumption that deception, even in reliable sources, should reduce operational confidence; an interpretation that is consistent with prior intelligence literature on cognitive bias and trust calibration (Heuer, 1999; Chen et al., 2023).

Further, the ability to produce a distribution of scenario probabilities across multiple sources allowed the TSSP model to reflect uncertainty more realistically than the rule-based systems (see Tables 13, 14B). Unlike binary thresholding, which can obscure diversity in behavioral variation, the continuous scale permitted risk-weighted planning. For instance, sources with high predicted reliability but non-negligible deception scores generated moderate scenario probabilities, prompting cautious engagement strategies. In contrast, sources with both high reliability and low deception significantly increased the likelihood of scenario selection, thereby, given priority in the tasking algorithm. This modeling approach affirms that ML-informed scenario generation contributes directly to more granular and risk-sensitive planning. In intelligence environments where incomplete information and deceptive behavior are common, such probabilistic modeling enables adaptive planning under uncertainty, which is a common challenge in the intelligence domain (Treverton and Gabbard, 2008; Bose, 2008). Equally, the TSSP framework, which is grounded in this behavioral realism, outperformed baseline heuristics in both expected cost and mission success rate.

### 5.3 TSSP optimization and resource efficiency

The TSSP model demonstrated robust optimization performance, returning feasible and optimal allocations across all defined behavioral scenarios. The convergence of the solver under constraints derived from ML predictions, such as task success rates, deception risk, and corroboration scores, confirmed the model's practical viability in real-world intelligence environments marked by uncertainty and limited observability.

The most notable findings were the consistency of the model to handle behavioral indicators. The optimisation outputs showed nearly uniform tasking across sources with high reliability and low deception scores, indicating the model's preference for sources with favorable behavioral profiles. This homogeneity in tasking patterns reflects the ability of the model to balance expected utility with risk in a principled way, consistent with stochastic optimisation theory (Birge and Louveaux, 2011). When bench-marked against a rule-based system, which ordinarily applies deterministic thresholds to behavioral features, the TSSP model yielded substantial operational gains. Particularly, it achieved a 16.8% reduction in expected tasking costs and a 19.3% improvement in mission success rate. These improvements illustrate the superiority of probabilistic planning over rigid heuristics, particularly in environments characterized by incomplete or deceptive information (Treverton and Gabbard, 2008). The reduction in tasking cost can be attributed to the ability of the model to de-prioritize high-deception or low-reliability sources early in the decision process, thereby conserving resources. Simultaneously, the increase in mission success rate suggests more effective source engagement and alignment with mission objectives.

Practically, these findings indicate that the TSSP model did not merely optimize on paper but produced operationally actionable plans that respected behavioral diversity and adapted dynamically to risk profiles. By integrating machine learning derived probabilities, the model leveraged predictive intelligence to simulate potential future states, that is, the behavioral scenario that each source might present, and incorporated this foresight into the present tasking decisions. This capability distinguishes TSSP from static or threshold-based systems, which apply fixed decision rules regardless of context. Whereas robust optimization assumes worst-case uncertainty and naturally provides conservative outcomes (Ben-Tal et al., 2009), and Bayesian networks, which are primarily suited for probabilistic inference and diagnostics rather than sequential decision-making or large-scale optimization (Lucas et al., 2004), TSSP provides a flexible, risk-sensitive approach.

### 5.4 Integration with ML pipeline

The integration of ML with the TSSP model produced a cohesive framework capable of enhancing task allocation decisions within intelligence operations. This hybrid approach bridged the predictive power of supervised learning with the prescriptive strength of optimization modeling, resulting in a system that not only forecasts behavioral outcomes but also acts upon them in a structured, risk-aware manner.

Central to this integration was the translation of ML-driven behavioral outputs, such as deception scores, reliability estimations, and classification confidence, into scenario probabilities. These probabilities served as the stochastic inputs for the TSSP model, allowing the decision framework to simulate and prepare for multiple possible behavioral conditions across intelligence sources. This process shifted task allocation from a reactive, rule-bound procedure to a proactive, data-informed planning mechanism that is capable of anticipating source behavior and allocating resources efficiently. The results validate this integrated approach in three critical dimensions. First, it led to resource optimization, with reduced tasking costs indicating more judicious allocation of analyst and handler time. Second, it supported risk mitigation, with the model avoiding high-risk sources by internalizing deception indicators into its probabilistic planning. Third, it improved mission throughput, a key operational metric measured by increased task success rates. This aligns with broader findings in intelligence analytics, where the combination of ML and operations research has been shown to improve responsiveness and strategic alignment (Chandola et al., 2009; Willard et al., 2022). The design also facilitates ongoing updates as new behavioral data becomes available, enabling continuous model refinement and scenario recalibration, a feature critical in a dynamic intelligence environment (Treverton and Gabbard, 2008).

### 5.5 Sensitivity and resource efficiency

The sensitivity analysis revealed that the TSSP model consistently outperformed the rule-based system across variations in tasking cost and success rate thresholds. As shown in Figure 10, the TSSP maintained lower tasking costs even at elevated cost levels, remaining below the $1,000 benchmark. At baseline, it achieved a 16.8% cost reduction ($832 vs. $1,000). This indicates that the model adapts efficiently under budget constraints by prioritizing high-value, reliable sources. Similarly, mission success rates improved under the TSSP model across all tested scenarios. At the baseline threshold, it achieved 83.5% success compared to 70% in the rule-based system, an increase of 19.3%. This gain reflects the model's ability to optimize assignments based on predicted reliability and deception scores. Handler resource use was also streamlined through optimized task distribution, enhancing operational efficiency. This confirms that TSSP not only improves source performance management but also sustains robustness under uncertainty.

### 5.6 Comparative evaluation of baseline approaches

The results show that while standalone ML models achieved high predictive accuracy, their lack of integration with optimisation limited operational impact. The deterministic model, though benefiting from an assumption of perfect foresight, was constrained by static allocation rules, yielding lower expected utility and higher regret. In contrast, the ML–TSSP framework leveraged XGBoost predictions within a scenario-based planning process, enabling more adaptive and efficient resource allocation. Its higher expected utility and lower regret demonstrate improved resilience and decision quality under uncertainty, supporting the case for integrating predictive modeling with stochastic optimisation in intelligence operations.

### 5.7 Limitations and areas for further study

While the ML–TSSP framework outperformed all baselines in the simulations, several limitations should be noted. First, the heuristic-based evaluation baseline could not be assessed quantitatively due to the absence of consistent, measurable operational data; it was therefore retained as a qualitative reference, which limits the completeness of direct numerical comparisons. Second, the values used for predicting deception and reliability were based on assumptions, not real-world data. In the future, it would be better to update the model using actual field feedback, such as task reviews or performance reports, to make the predictions more accurate.

Another concern involves how the CI flag was modeled. In this study, it functioned as a simple binary indicator, either flagged or not. However, CI issues in practice often involve complex variables of trust, partial compromise, or ongoing investigations. Incorporating a probabilistic or fuzzy logic approach, as suggested by (Jajodia et al. 2016) on adversarial reasoning, could improve accuracy and decision quality. Another limitation involves the use of SMOTE for addressing class imbalance during model training. Although this method helps avoid model bias toward dominant behavioral types, it may distort the natural frequency of rare but operationally significant traits like consistently cooperative or high-performing sources. This should be considered during interpretation. Lastly, the framework was designed with tactical operations in mind. Extending the model to strategic intelligence coordination, such as integrating signals, imagery, and human intelligence sources across units, could present a valuable addition in future exploration.

## 6 Conclusion and recommendations

This study tackled a key problem in intelligence operations: how to manage the performance of human sources when their behavior is uncertain and the stakes are high. By combining supervised machine learning with two-stage stochastic programming, the project introduced a practical framework for predicting how sources might behave and using that information to guide tasking decisions more effectively. The results showed that this combined approach works. The machine learning models were able to forecast source reliability and deception risk with strong accuracy, and these forecasts were converted into probabilities used in the optimization model. The model, in turn, helped improve how resources were allocated. Compared to traditional rule-based methods, the framework cut tasking costs by nearly 17% and improved mission success by over 19%. It also proved capable of adjusting across different types of source behavior, focusing support on the most dependable while avoiding high-risk actors. In practical application of intelligence operations, this means intelligence managers can make better decisions earlier, with the flexibility to adjust if a source is less reliable than expected. While the study relied on simulated data and simplified some variables like the counterintelligence flag, it provides a solid foundation for more advanced, data-informed approaches to the management of HUMINT sources. Equally, the framework could be further developed using online learning techniques, allowing the system to continuously update its predictions as new performance data becomes available from field reports, task outcomes, or debriefs. Similarly, reinforcement learning could be integrated to refine tasking policies over time, rewarding decision paths that lead to reliable reporting and penalizing for risk or misinformation-prone initiatives.

### 6.1 Recommendations

The following are some of the notable recommendations for consideration based on this study;

i) Future models should incorporate real-world feedback mechanisms such as post-operation evaluations, source debriefings, and handler assessments. This would allow the machine learning component to continuously learn from operational outcomes, improving the accuracy and reliability of behavioral predictions over time.
ii) The framework should be extended to integrate multiple intelligence streams beyond HUMINT, such as signals intelligence (SIGINT), open-source intelligence (OSINT), and geospatial intelligence (GEOINT). This would enable a more comprehensive approach to tasking by capturing interdependencies across sources and domains.
iii) Variables such as the counterintelligence (CI) flag should be modeled using fuzzy logic or probabilistic methods instead of binary indicators. This would reflect the complex, often uncertain nature of real-world CI assessments and allow for more flexible decision-making under ambiguity.
iv) The framework should be tested in simulation environments or operational war games prior to deployment. These controlled trials would help evaluate system robustness, user interpretation of model outputs, and the practical impact on decision cycles under realistic conditions.
v) It is essential to develop user-friendly interfaces that translate model outputs into actionable insights for intelligence managers. Such interfaces should support visualization of scenario probabilities, trade-off comparisons, and include options for manual override to preserve the role of expert judgment.

## Data Availability

The raw data supporting the conclusions of this article will be made available by the authors, without undue reservation.
